# The Effect of Naturally Acquired Immunity on Mortality Predictors: A Focus on Individuals with New Coronavirus

**DOI:** 10.3390/biomedicines13040803

**Published:** 2025-03-27

**Authors:** Mónica Queipo, Jorge Mateo, Ana María Torres, Julia Barbado

**Affiliations:** 1 Autoimmunity and Inflammation Research Group, Río Hortega University Hospital, 47012 Valladolid, Spain; mqueipo@saludcastillayleon.es; 2 Cooperative Research Network Focused on Health Results—Advanced Therapies (RICORS TERAV), 28220 Madrid, Spain; 3 Medical Analysis Expert Group, Institute of Technology, University of Castilla-La Mancha, 13001 Cuenca, Spain; 4 Medical Analysis Expert Group, Instituto de Investigación Sanitaria de Castilla-La Mancha (IDISCAM), 45071 Toledo, Spain; 5 Internal Medicine, Río Hortega University Hospital, 47012 Valladolid, Spain

**Keywords:** immunity, COVID-19, predictors, mortality, machine learning, Random Forest

## Abstract

**Background/Objectives**: The spread of the COVID-19 pandemic has spurred the development of advanced healthcare tools to effectively manage patient outcomes. This study aims to identify key predictors of mortality in hospitalized patients with some level of natural immunity, but not yet vaccinated, using machine learning techniques. **Methods**: A total of 363 patients with COVID-19 admitted to Río Hortega University Hospital in Spain between the second and fourth waves of the pandemic were included in this study. Key characteristics related to both the patient’s previous status and hospital stay were screened using the Random Forest (RF) machine learning technique. **Results**: Of the 19 variables identified as having the greatest influence on predicting mortality, the most powerful ones could be identified at the time of hospital admission. These included the assessment of severity in community-acquired pneumonia (CURB-65) scale, age, the Glasgow Coma Scale (GCS), and comorbidities, as well as laboratory results. Some variables associated with hospitalization and intensive care unit (ICU) admission (acute renal failure, shock, PRONO sessions and the Acute Physiology and Chronic Health Evaluation [APACHE-II] scale) showed a certain degree of significance. The Random Forest (RF) method showed high accuracy, with a precision of >95%. **Conclusions**: This study shows that natural immunity generates significant changes in the evolution of the disease. As has been shown, machine learning models are an effective tool to improve personalized patient care in different periods.

## 1. Introduction

When a sufficient number of individuals are immunized against a pathogen, the probability of transmission between the infected and susceptible population decreases due to the interruption of the chain of transmission [1]. In the case of a contagious, direct contact-transferable pathogen that induces long-term immunity, this is maximized if the population has a random pattern of interaction [2]. Diseases such as rubella, measles or pertussis are under control, and others such as smallpox are even eradicated today thanks to herd immunity, either by natural means or through vaccination strategies [3].

Natural immunity, which develops after infection with COVID-19, and vaccine-induced immunity, achieved through controlled exposure to viral antigens, are key mechanisms for protection against the virus. While natural immunity depends on factors like the initial viral load and may be less predictable, vaccines offer more uniform protection and mitigate the risks associated with severe disease [4]. Moreover, recent studies suggest that the combination of both types of immunity can provide more robust and long-lasting protection [5].

This concept gained significant visibility during the coronavirus disease-2019 (COVID-19) pandemic, as the need to achieve herd immunity became a critical objective to minimize the clinical and societal impact of the health emergency. Early estimates indicated that 70% of the population would need immunization to achieve this goal [6]. Data from populations with full vaccination schedules suggest that this threshold was reached between the third and fourth waves of the pandemic (June–September 2021) in regions, such as Europe, Oceania, the Middle East, and North America, while Asia and Africa reached these rates later, between late 2021 and mid-2022 [7].

Well before vaccination campaigns began, however, a substantial percentage of the population had already acquired natural immunity. Studying the characteristics of individuals infected during this period—before vaccination but after the first wave—is of profound clinical interest. Understanding their natural immune responses, the variability in disease severity, treatment protocols, and virus epidemiology provides invaluable insights for managing future outbreaks or similar health crises.

Severe cases of COVID-19 are characterized by an inflammatory response and a cytokine storm that affects all cells of the immune system [8], especially lymphocytes. This has been shown to cause a dysregulation that can lead to an uncontrolled immune response, lung tissue damage or even multi-organ failure [9]. The initial symptoms of the disease, as reported at the onset of the pandemic, included fever, dyspnea, pneumonia and a dry cough [10,11,12]. Nevertheless, differences in clinical presentation across pandemic waves have been observed, such as reduced intensive care unit (ICU) admission rates [13] or decreased mortality [14,15]. It is evident that this progression can be attributed, firstly, to a heightened comprehension of the disease, facilitated by scientific research and the implementation of social policies. However, it is also associated with the modification of immunological characteristics at both the individual and population levels, owing to the progressive development of immunity, as a growing number of individuals recover from the infection and achieve immunological protection.

Comparing COVID-19 with earlier respiratory infections caused by coronaviruses, such as severe acute respiratory syndrome coronavirus (SARS-CoV) (2002–2003) and Middle Eastern respiratory syndrome coronavirus (MERS-CoV) (2012), offers additional perspective. Both SARS-CoV and MERS-CoV share similarities with SARS-CoV-2 in transmission routes [16] and clinical features, though with distinct differences in transmission rates and global impacts [17]. Lessons learned from studies on these viruses provide a framework for analyzing COVID-19 data and underscore the utility of advanced analytical techniques such as machine learning (ML).

In the context of data analysis from a limited sample of patients during a pandemic, advanced data analysis techniques are increasingly being used. Among artificial intelligence (AI) tools, machine learning (ML) has seen a surge in utilization in studies dealing with voluminous datasets. This branch of AI and computational analysis entails the employment of data and algorithms that emulate human information processing capabilities while enhancing efficiency [18]. Its efficacy has been demonstrated in several areas of knowledge, including biology [19] or medicine [20,21,22]. Furthermore, these machine learning tools have been extensively utilized in research related to the novel coronavirus, SARS-CoV-2, particularly in the study of mortality [23,24] and/or severity [25,26], the study of populations with specific characteristics (smokers [27], cancer patients [28,29], etc.) and biomarker analysis [30,31].

A notable benefit of leveraging ML models over conventional statistical tools is their capacity to generate precise predictions while exhibiting high levels of scalability and adaptability. This capability enables the identification of patterns within voluminous datasets. This is particularly relevant in the context of a public health problem that was not only unknown in its early stages but has shown a remarkable evolution over different time periods. Consequently, the objective of this study is to identify key predictors of mortality risk in a cohort of patients hospitalized for COVID-19 during a period when vaccines were not yet available but immunity obtained by natural means was present.

For this purpose, the specific ML model to be validated is the Random Forest (RF). This method might be utilized systematically as a risk evaluation tool in any population, enabling the derivation of conclusions in a relatively brief time frame with data from a limited number of patients (150–300). This approach would enable the development of customized protocols for diverse populations, such as health centers or cities, with the objective of optimizing resource utilization and ensuring a highly personalized healthcare experience.

## 2. Materials and Methods

### 2.1. Data Source and Description

The clinical data used in this study were taken from the electronic medical record system of the Río Hortega University Hospital in Valladolid (Spain). Data from 363 patients hospitalized with polymerase chain reaction (PCR)-confirmed COVID-19 infection between the beginning of the second wave (2 May 2020) and the end of the fourth wave (10 June 2021) were obtained from this platform. The information used in this study corresponds to the patients’ hospital stay from the time of admission to the emergency department until discharge from the hospital. Each patient was given an anonymous code to protect their privacy, and all patients gave informed consent. This study was conducted according to the principles of Helsinki and was approved by the Ethics Committee of the University Hospital of Rio Hortega.

Data were collected, retrospectively reviewed, and manually entered into a predesigned database. These data included demographics, comorbidities, chronic treatments, date of admission, date of discharge or death and cause, symptoms on admission, date of COVID-19 diagnosis and virus variant, if available, chest X-ray on admission, community acquired pneumonia severity scales (CURB-65), sequential organ failure assessment (SOFA), acute physiology and chronic health disease classification system (APACHE-II), Glasgow Coma Scale (GCS), vital signs, laboratory data, need for intensive care unit (ICU) admission and date (if applicable), need and type of ventilatory support, co-infections, nosocomial infections (specimen typing), complications during hospitalization, previous COVID-19 episodes, COVID-19 specific treatment, and number and type of COVID-19 vaccines received.

The laboratory tests whose results were used in this study were performed at the same hospital center using the following instruments: DXH900 Beckman Coulter Diagnosis (Brea, CA, USA) for whole blood samples, AU5820 Beckman Coulter Diagnosis for serum biochemistry samples and Gem5000 Werfen (Barcelona, Spain) for blood gasometry analysis. The data were entered into the aforementioned hospital electronic data storage system prior to their use in this study. The laboratory parameters considered relevant to this study were the following: leukocytes, neutrophils, lymphocytes, monocytes, eosinophils, basophils, erythrocytes, hemoglobin, hematocrit, mean corpuscular volume (M.C.V.), platelets, D-dimer, prothrombin activity (PT), international normalized ratio (I.N.R.), activated partial thromboplastin time (aPTT), aPTT ratio, derived fibrinogen, sodium, potassium, chloride, glucose, urea, creatinine, estimated glomerular filtration rate (CKD-EPI 2009), alanine aminotransferase (ALT/GPT), aspartate aminotransferase (AST/GOT), gamma glutamyl transferase (GGT), total bilirubin, alkaline phosphatase, lactate dehydrogenase (LDH), phosphate, C-reactive protein, procalcitonin, albumin, pH, pCO_2_, pO_2_, HCO_3_, FIO_2_, pO_2_/FIO_2_, O_2_ gradient Aa and lactate.

These variables were selected after a thorough literature review to identify the most critical factors during the pandemic. The focus was on those that could significantly aid in evaluating disease severity, immune response, and associated risk factors. Additionally, the chosen parameters were those that could be quickly and routinely measured both at the time of hospital admission and throughout the patient’s stay in the hospital.

### 2.2. Machine Learning Methods

In this study, the Random Forest algorithm was developed, an ensemble method that employs the bagging aggregation approach to construct multiple decision trees independently, thereby reducing variance and enhancing the robustness of the model. Random Forest is based on bootstrap sampling of the training data to build a set of independent trees, where each tree is trained on a random sample of the original dataset. Furthermore, at each tree node, a random subset of features is selected instead of considering all variables, introducing an additional source of randomness and minimizing correlation between the trees, thereby increasing the accuracy of the ensemble [32,33].

During the training process, each tree makes decisions independently, and the results are combined using a majority voting scheme for classification. Feature importance is calculated by analyzing the decrease in accuracy or Gini index when the values of a feature are randomly permuted, thereby identifying the most relevant variables for the model. Finally, the model’s performance was evaluated using specific metrics such as accuracy, sensitivity, specificity, and the area under the receiver operating characteristic (ROC) curve (AUC), enabling validation of its predictive capability and generalization to unseen data.

In the proposed Random Forest algorithm, a set of decision trees {*T*_1_, *T*_2_, …, *T*_m_} is constructed using a bagging aggregation approach. To build each tree *T_i_*, the following steps were performed:

Bootstrap sampling: Given a training dataset with *n* observations and *p* features, a subset of data *Di* is generated by selecting n random samples with replacement from the original dataset. This sampling technique allows certain data points to appear multiple times in *Di*, while others may not appear at all.

Random feature selection: At each node of every tree, instead of evaluating all *p* features, a random subset of *k* features is selected, where k=√p. This reduces the correlation between individual trees, thereby increasing the model’s generalization ability.

Splitting criterion at each node: Each node is split according to an impurity reduction criterion, which can be either entropy or the Gini index for classification. In our study, we use the Gini index. The Gini impurity *G* of a node with a proportion *pk* of elements belonging to class *k* is defined as:

$$G=1-\sum_{k=1}^{K}p_{k}^{2}$$

Combination of trees: Once the trees are trained, the prediction of the Random Forest is obtained through aggregation. For a set of trees {*T*_1_, *T*_2_, …, *T*_m_}, the final prediction $\hat{y}$ is calculated using majority voting:

$$\hat{y}=mode\{T_{1}\left(x\right),\;T_{2}\left(x\right),\ldots ,T_{m}\left(x\right)\}$$

Feature importance: The importance of each feature was measured by evaluating the change in the splitting criterion when the feature was randomly permuted in the dataset. For importance based on the Gini index, if permuting a specific feature increased the impurity of the tree nodes, that feature was considered important.

Model optimization and tuning: In order to improve the predictive performance of the Random Forest model and avoid overfitting, hyperparameter tuning was performed using a Bayesian optimization strategy. This approach was preferred over grid search due to its efficiency in navigating large parameter spaces with fewer evaluations. The optimization was carried out with a fivefold cross-validation strategy applied to the training data. The hyperparameters included the number of trees in the forest (*n*_estimators), the maximum depth of the trees (max_depth), the minimum number of samples required to split an internal node (min_samples_split), and the number of features considered at each split (max_features). The optimization objective was to maximize the average cross-validated AUC. This procedure allowed us to determine an optimal configuration that balances model complexity and generalization capacity, minimizing the risk of overfitting.

Model evaluation: The model’s performance was evaluated using metrics, such as accuracy, sensitivity, specificity, and the AUC.

In this study, the proposed method underwent extensive evaluation, comparing it with various machine learning techniques for classifying COVID-19 patients. The algorithms included in the comparative analysis were Gaussian Naive Bayes (GNB) [34,35], k-Nearest Neighbors (KNNs) [36,37], Bayesian Linear Discriminant Analysis (BLDA) [38,39], Support Vector Machines (SVM) [40], decision trees (DT) [41,42], and the novel system developed in this study, Random Forest (RF) [32,43,44]. The implementation and evaluation of the models were conducted using MATLAB’s Statistics and Machine Learning Toolbox (version 2024a).

**Figure 1:**
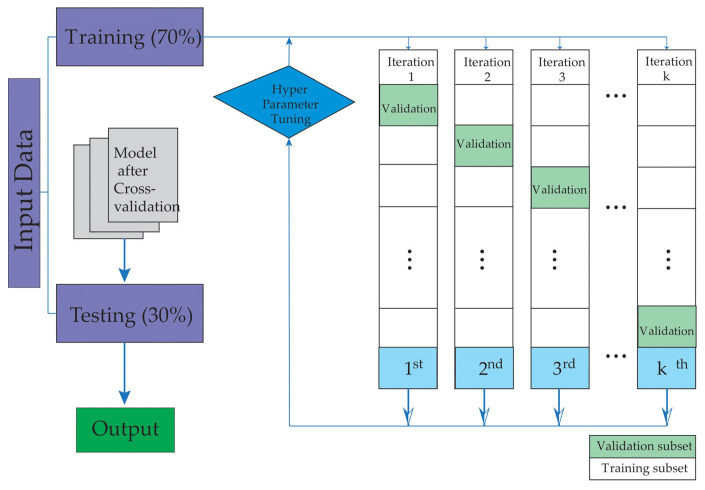
The figure illustrates the training and validation stages used in this study.

To mitigate overfitting, a fivefold cross-validation strategy was applied. The data were split into two subsets, assigning 70% for training and 30% for testing, ensuring the independence of patient groups in each set. Figure 1 schematically illustrates the study workflow, which begins with patient selection and database creation, followed by the training and validation phases of the machine learning models.

## 3. Results

A total cohort of 363 patients was studied, corresponding to the second (135 patients), third (115 patients) and fourth (113 patients) waves of the pandemic, when mass vaccination campaigns had not yet begun. The study identified 40 deceased patients, resulting in an overall mortality rate of 11%.

The medical treatments and protocols used during the second to fourth waves of COVID-19 were stable during this period and followed hospital, local health and ministry of health protocols, based on WHO indications.

The information obtained is related to the patient’s previous condition, the time of admission, and also the evolution of the patient during the hospital stay. More detailed data on demographics, comorbidities, chronic treatments, symptoms on admission, ICU stay, need for ventilatory support or complications during hospital stay, among others, are shown in Table 1, Table 2, Table 3 and Table 4.

Several ML methods were used to identify risk patterns in a population with confirmed COVID-19 infection. The goal was to determine which algorithm provided the best predictive results. This study presents performance metrics for these ML methods, including balanced accuracy, recall, specificity, precision, Mathew’s correlation coefficient (MCC), F1 score, kappa, AUC, and degenerated Youden’s index (DYI), as shown in Table 5 and Table 6.

The data clearly indicate that the proposed method, RF, is the one with the highest acquisition and recall value. The RF algorithm consistently provides a positive predictive value of greater than 95%, demonstrating consistent performance.

**Figure 2:**
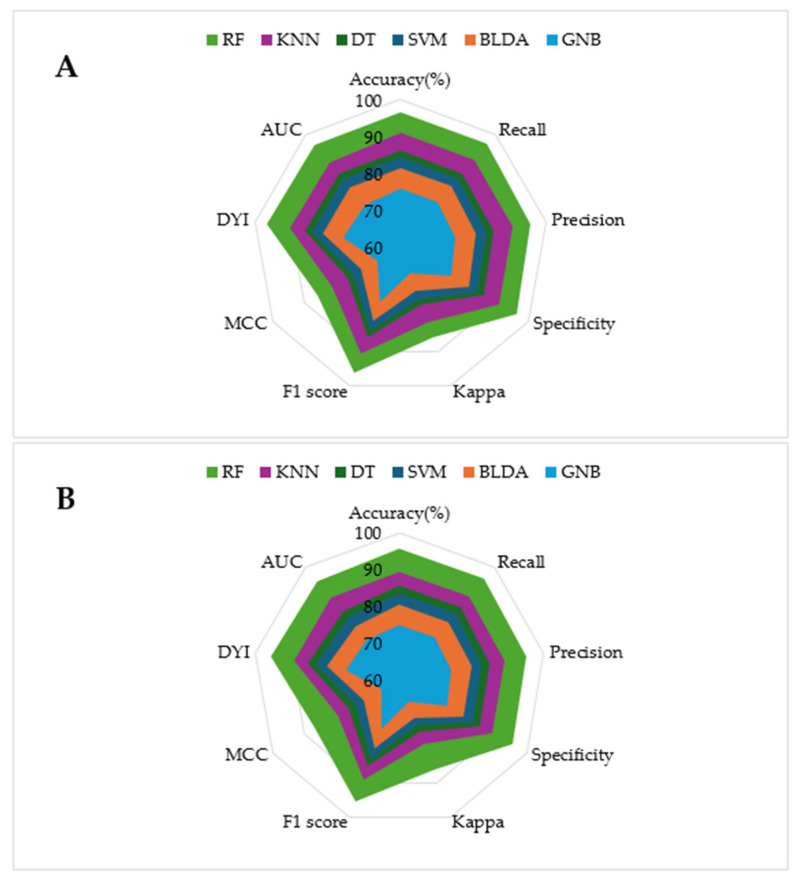
The figure shows the radar plots comparing multiple performance metrics for specific models. Each axis represents a key performance metric. The radial axis ranges from 0 (minimum relevance) to 100% (maximum relevance). (**A**) Training phase. (**B**) Test phase.

The model training subset and the test subset both present high scores for all the metrics, with slightly lower scores for the test subset. This consistency is attributed to the algorithm reaching an optimal level of training without overfitting or underfitting. As shown in the radar plots in Figure 2, the RF model covers a larger area compared to the other methods tested, which is an example of a well-balanced model with high generalization capability, ensuring accurate outputs for new inputs.

**Figure 3:**
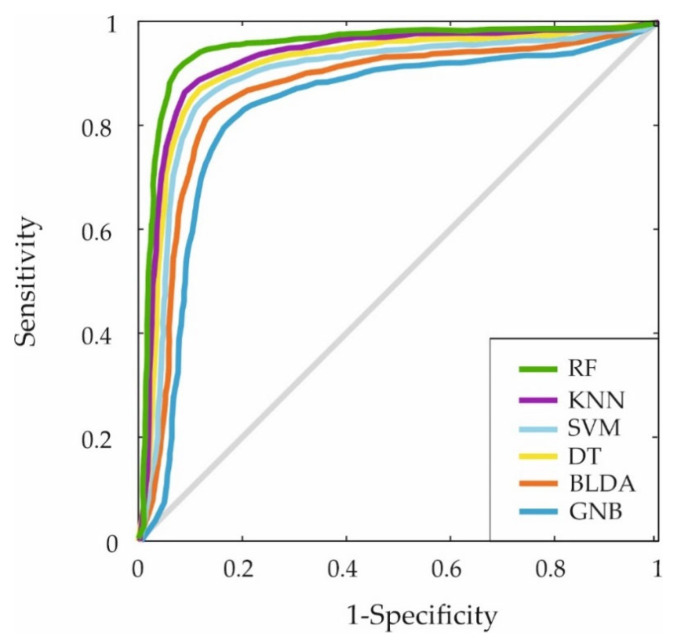
The figure shows the ROC curves for the six machine learning predictors. This curve shows the trade-off between sensitivity (true positive rate) and 1-specificity (false positive rate) for the ML models mentioned: RF, KNN, SVM, DT, BLDA and GNB.

Furthermore, the ROC curve was generated by plotting the sensitivity and specificity measures for each threshold to evaluate the classification capabilities of the different ML algorithms. The results are displayed in Figure 3. Again, the proposed RF-based system covers a larger area, indicating superior predictive accuracy.

**Figure 4:**
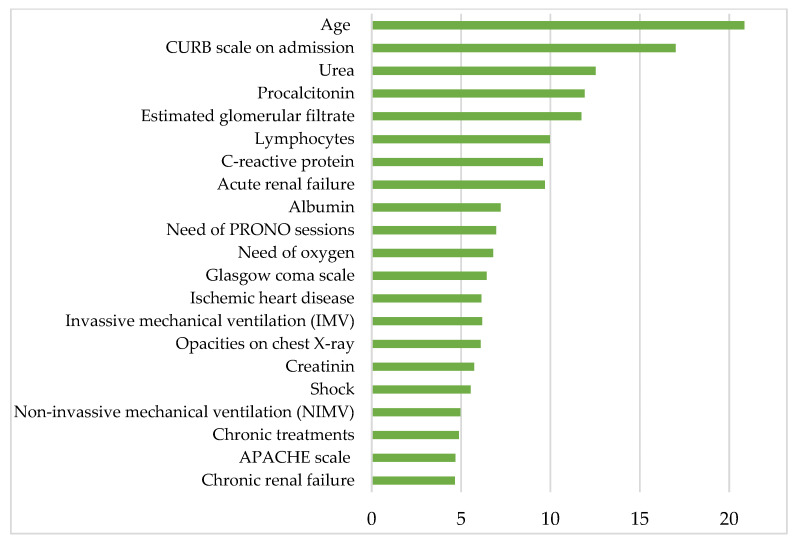
The histogram shows the 21 most relevant parameters contributing to the mortality in COVID-19 hospitalized patients on the *y*-axis. The *x*-axis represents the statistical weight or importance assigned of each parameter in predicting mortality. More details about the units in which the parameters are presented in Table 1, Table 2, Table 3 and Table 4.

Figure 4 shows the most clinically relevant parameters contributing to mortality in patients hospitalized for COVID-19 after the first wave and before mass vaccination, according to the proposed RF model. Listed in descending order of relevance, the aforementioned parameters are age, CURB-65 scale on admission, urea, procalcitonin, estimated glomerular filtrate, lymphocytes, C-reactive protein, acute renal failure, albumin, PRONO sessions, oxygen requirements, Glasgow Coma Scale, ischemic heart disease, invasive mechanical ventilation (IMV), creatinine, shock, noninvasive mechanical ventilation (NIMV), APACHE-II scale and chronic renal failure.

## 4. Discussion

Despite significant progress in the fight against COVID-19 and the time we have been coexisting with the virus after the global emergency, the pandemic remains a global concern. The emergence of new variants and the increase in cases during certain periods underscore the need to continue to research and improve our response strategies. In addition, the pandemic has highlighted weaknesses in health systems and the importance of researching more effective interventions and protocols to provide higher-quality care.

In this study, the analysis of the most relevant parameters for predicting the risk of mortality in hospitalized COVID-19 patients is organized into different categories for clarity and understanding. These categories include demographic factors, clinical features observed on admission, laboratory parameters, and variables monitored during hospitalization. This categorization highlights the complementary roles these parameters play in predicting mortality and enhances the study’s contribution to clinical decision making.

The most relevant parameters for predicting the risk of mortality in this group of hospitalized patients with COVID-19 during the period from the second to the fourth wave of the pandemic were identified. At that time, there was some immunity following infections in previous waves, and the population had not yet been vaccinated with more than one dose or had not yet been vaccinated at all. Among the key variables identified, the most powerful parameters were those that could be obtained at the time of hospital admission, either previous comorbidities or the clinical features assessed in the emergency department.

Thus, the strongest predictor among demographic factors is age, a conclusion common to many studies [45,46]. It should be emphasized that, although the majority of the patients in this cohort were older adults, this does not diminish the importance of age as a predictive factor. Rather than merely reflecting the higher mortality commonly associated with older populations, age itself plays a crucial role in the progression and outcome of COVID-19. This is supported by extensive evidence in the medical literature highlighting the physiological and immunological changes associated with aging that increase susceptibility to severe disease. To ensure the robustness of the analysis, statistical techniques were employed, including multivariate models, to isolate the independent contribution of age and confirm its predictive power while accounting for the influence of other clinical and pathologic parameters.

Similar trends were observed in SARS-CoV and MERS, where older age was also a significant risk factor for mortality, although the overall case fatality rates differed markedly, SARS-CoV at ~10% and MERS-COV at ~35%, compared to COVID-19′s lower fatality rate but higher transmissibility [47]. However, Philipps and Carver [48] concluded that it is not so much the age itself but what comes with advanced age that is a risk factor for mortality, namely comorbidities, chronic treatments and social situation. The results of this study support their theory, in that some of the factors that emerge as strong predictors of mortality are related to chronic diseases, such as ischemic heart disease and chronic renal failure.

The CURB-65 scale, a hallmark in the results, includes age as a variable. This scale has been evaluated in other studies as an adequate predictor of mortality in patients prior to hospitalization with COVID-19 pneumonia [49,50,51], and it is also commonly used to determine the severity in patients with any type of pneumonia. In addition to demographic factors, clinical features such as those included in the CURB-65 scale, such as urea levels or low level of consciousness, have considerable predictive power for mortality. In this study, the CURB-65 scale is a good parameter, and since it can be determined at the time of admission, this information can be used to perform rapid and efficient triage. Furthermore, a comparison with other viruses reveals that this scale, which focuses on clinical severity, has also been used to effectively manage pneumonia-like symptoms [52,53].

Laboratory parameters obtained at the time of hospitalization, such as urea levels, C-reactive protein, procalcitonin, albumin and/or lymphocyte count, are also good predictors of mortality according to the model used. Mohammadi et al. studied more than 1000 patients and concluded that elevated creatinine and urea levels were associated with poor prognosis in patients with COVID-19 [54]. Other studies, such as those of Al-Shajlawi et al. [55] or Singh and Singh [56], also include elevated C-reactive protein and procalcitonin levels, low albumin levels and low lymphocyte count as good predictors of mortality. Elevated urea levels, together with low lymphocyte levels, may be related to a severe systemic inflammatory response and consequent inflammation. C-reactive protein and procalcitonin are known markers of inflammation and sepsis, and their elevation reflects the severity of the inflammatory response. On the other hand, albumin, a protein produced by the liver, is normally decreased in states of chronic inflammation and physiological stress due to its role in the acute-phase response. A low lymphocyte count, or lymphopenia, may indicate immune dysfunction, which is common in severe viral infections such as COVID-19. All of these data would be consistent with a state of advanced inflammation and organ damage, explaining the association with an increased risk of mortality.

Moreover, this study shows that certain in-hospital monitoring variables, including acute renal failure and shock, have predicting significance. These belong to the category of monitoring parameters, which track the disease progression during hospitalization and provide critical insights into mortality risk. The study by Yüksel et al. [57] associates the aforementioned imbalances in laboratory parameters with the development of acute renal failure. At the same time, studies such as that by Qureshi et al. [58] have shown that non-surviving patients have a higher incidence of septic shock, among other pathologies. This underscores the importance of continuous monitoring and early intervention in COVID-19 patients with these alterations.

Additional monitored variables, such as the need for oxygen therapy, prone positioning sessions (PRONO), or mechanical ventilation, have also shown predictive significance for mortality risk as the disease progresses. Studies show they hold predictive significance for mortality risk as the disease progresses during ICU stay [59]. In turn, the APACHE-II scale has been shown to be a good predictor of mortality in some studies, although there are contradictions in this regard [59,60,61,62]. These include physiologic and biochemical parameters assessed on admission to the ICU that reflect the systemic response to severe physiologic stress common in severe cases of COVID-19. The higher relevance of APACHE-II in our study may be related to its ability to better capture multiorgan dysfunction in the early stages, providing a more robust prediction of mortality.

The data management approach used in this study involved an RF machine learning model, which was based on either bootstrap aggregation or bagging. This model has exhibited several advantageous characteristics, including high generalizability, stability and interpretability, along with a low risk of overfitting. Other studies have used the same method to analyze diseases, such as cardiovascular disease [63], interstitial lung disease [64], breast [65] or pancreatic cancer [66], autoimmune diseases [63] or neurological diseases [67] or neurodegenerative diseases [68]. The model can, therefore, be used as a strategic tool to quickly address clinical questions with relatively little data.

The application of the aforementioned model in the analysis of the variables with the most significant influence on the prediction of mortality in the cohort of hospitalized patients with COVID-19 during the second and third waves reveals a discrepancy in the results compared to those of analogous studies conducted in the first wave [11,12,13,69,70,71]. In these studies, patients had not yet developed any form of immunity. In contrast, the present study demonstrates that once a population achieves a certain level of immunity, additional predictive variables can be obtained at the time of admission. Furthermore, during the patient’s hospital stay, variables related to the stay itself were identified, and these variables can be used in decision making.

## 5. Conclusions

After the first wave of the recent COVID-19 pandemic, the size of the population that has overcome the infection and developed natural immunity is increasing. Therefore, it is crucial to study how natural immunity affects the evolution of the disease in order to improve general knowledge about it and improve preparedness for potential new health challenges.

The present study demonstrates that predictive variables for mortality risk, and, therefore, severity, can be identified in a given population at the time of hospital admission using an RF machine learning model. In this cohort, the CURB-65 scale and age were the strongest predictors of mortality, along with other laboratory tests. These results underscore the need to emphasize not only the early patient assessment but also the personalized analysis of the population and situation, given their capacity for change.

The present study shows that the increase in the number of immunized patients, even in the absence of herd immunity, generates a significant modification in the evolution of the disease, as well as the mortality risk parameters. This underscores the necessity for a more customized approach to patient care, employing distinct protocols tailored to specific groups and temporal periods. In this regard, the employment of machine learning tools emerges as a highly valuable and efficient resource.

## Figures and Tables

**Table 1:** Parameters related to patient history and demographics.

	Deceased (40 Patients, 11%)		Alive (323 Patients, 89%)		Global (363 Patients, 100%)	
	(*n*)	(%)	(*n*)	(%)	(*n*)	(%)
**Sex**						
Men	33	83	198	61	231	64
Women	7	18	125	39	132	36
**Comorbidities**						
Hypertension	24	60	144	45	168	46
Diabetes mellitus	10	25	68	21	78	21
Ischemic cardiopathy	7	18	17	5	24	7
Cardiac insufficiency	1	3	6	2	7	2
COPD (Chronic emphysema/bronchitis)	1	3	17	5	18	5
Asthma	1	3	17	5	18	5
Dyslipidemia	16	40	100	31	116	32
Coagulopathies	7	18	34	11	41	11
Renal insufficiency	5	13	19	6	24	7
Active tumors	1	3	16	5	17	5
Immune-mediated diseases	2	5	38	12	40	11
Overweight	1	3	4	1	5	1
Obesity	3	8	30	9	33	9
**Chronic treatments**	32	80	183	57	215	59
Antihypertensives	21	53	115	36	136	37
Beta-blockers	10	25	38	12	48	13
Diuretics	8	20	37	11	45	12
Antidiabetics	8	20	58	18	66	18
Anti-aggregants	11	28	33	10	44	12
Anticoagulants	6	15	34	11	40	11
Lipid lowering agents	15	38	90	28	105	29
Chemotherapy	1	3	2	1	3	1
Immunosuppressive chronic treatment	3	8	21	7	24	7
Antiretrovirals	0	0	1	0	1	0
Antivirals	0	0	1	0	1	0
**Vaccination status**						
1 dose	2	5	15	5	17	5
2 doses	0	0	6	2	6	2
3 doses	0	0	0	0	0	0
4 doses	0	0	0	0	0	0

**Table 2:** Clinical characteristics of patients at the time of hospital admission.

	Deceased (40 Patients, 11%)		Alive (323 Patients, 89%)		Global (363 Patients)	
	(*n*)	(%)	(*n*)	(%)	(*n*)	(%)
**Symptoms on admission**						
Dyspnea	19	48	172	53	191	53
Chest discomfort	1	3	44	14	45	12
Cough	21	53	202	63	223	61
Rhinorrhea	0	0	10	3	10	3
Loss of smell (anosmia)	0	0	49	15	49	13
Loss of taste (ageusia)	2	5	48	15	50	14
Odynophagia	0	0	12	4	12	3
Myalgia	4	10	57	18	61	17
Fever	26	65	225	70	251	69
Dysthermia	3	8	27	8	30	8
Headache	2	5	33	10	35	10
Nausea/Vomiting	5	13	47	15	52	14
Diarrhea	3	8	65	20	68	19
Asthenia	7	18	101	31	108	30
Confusion	9	23	8	2	17	5
Dizziness	2	5	19	6	21	6
Sputum	6	15	45	14	51	14
**Diagnosis on admission**						
Respiratory distress	6	15	21	7	27	7
Acute respiratory insufficiency	20	50	127	39	147	40
Multiorgan failure	2	5	0	0	2	1

**Table 3:** Patient characteristics associated with hospitalization.

	Deceased (40 Patients, 11%)		Alive (323 Patients, 89%)		Global (363 Patients)	
	(*n*)	(%)	(*n*)	(%)	(*n*)	(%)
ICU admission	21	53	61	19	82	23
Invasive mechanical ventilation (IMV)	19	48	56	17	75	21
High Flow Nasal Cannula (HFNC)	4	10	29	9	33	9
Non-invasive mechanical ventilation (NIV)	12	30	30	9	42	12
Prone sessions	12	30	28	9	40	11
Extracorporeal membrane oxygenation (ECMO)	1	3	4	1	5	1
At least one previous episode of COVID	1	3	2	1	3	1
**Nosocomial infections**						
Viral	0	0	2	1	2	1
Bacterial	11	28	30	9	41	11
Fungal	3	8	3	1	6	2
**Complications during hospital stay**						
Acute renal failure	13	33	16	5	29	8
Cardiac	9	23	15	5	24	7
Arrhythmias	9	23	15	5	24	7
Gastrointestinal	5	13	22	7	27	7
Increased transaminases	5	13	10	3	15	4
Ileus	0	0	9	3	9	2
Mesenteric ischemia	0	0	1	0	1	0
Subocclusion	0	0	2	1	2	1
Neurological	3	8	19	6	22	6
Delirium	1	3	15	5	16	4
Encephalopathy	1	3	0	0	1	0
Peripheral neuropathy	1	3	4	1	5	1
Coagulopathies	9	23	14	4	23	6
Deep vein thrombosis	0	0	2	1	2	1
Pulmonary thromboembolism	3	8	7	2	10	3
Stroke	1	3	2	1	3	1
Bleeding	5	13	3	1	8	2
Respiratory distress (ARDS)	15	38	36	11	51	14
Shock	5	13	1	0	6	2
**Treatment for COVID-19 during hospital stay**						
Oxygen	32	80	173	54	205	56
Corticosteroids	26	66	142	44	168	46
Rendesivir	2	5	26	8	28	8
Ceftriaxone	21	53	142	44	163	45
Azithromycin	15	38	111	34	126	35
Heparin	8	20	112	35	120	33
**Cause of death (if applicable)**						
Multiorgan failure	17	43	0	0	17	5
Respiratory distress	1	3	0	0	1	0
Respiratory failure	11	28	0	0	11	3

**Table 4:** Numerical baseline clinical characteristics of patients. Mean and standard deviation (SD) were calculated.

	Deceased (40 Patients, 11%)		Alive (323 Patients, 89%)		Global (363 Patients)	
	Mean	SD	Mean	SD	Mean	SD
Age	79.53	10.92	64.40	16.18	66.08	16.37
Leucocytes (×10^3^ µL)	7.99	5.12	6.96	4.86	7.07	4.90
Lymphocytes (×10^3^ µL)	0.71	0.36	1.53	5.60	1.43	5.28
Neutrophils (×10^3^ µL)	6.42	5.23	5.05	3.80	5.20	4.00
Monocytes (×10^3^ µL)	0.50	0.29	0.61	0.81	0.60	0.77
Eosinophils (×10^3^ µL)	0.03	0.09	0.02	0.07	0.02	0.07
Basophils (×10^3^ µL)	0.14	0.74	0.02	0.04	0.03	0.25
Erythrocytes (×10^6^ µL)	4.40	0.68	4.72	0.70	4.69	0.70
Hemoglobin (g/dL)	13.41	1.87	13.79	1.83	13.75	1.83
Hematocrit (%)	39.62	7.00	41.52	5.36	41.30	5.59
M.C.V. (fL)	90.78	9.99	87.81	7.25	88.15	7.64
Platelets (×10^3^ µL)	163.52	80.76	186.62	79.00	184.02	79.42
Glucose (mg/dL)	144.83	62.46	138.84	62.98	139.50	62.86
Urea (mg/dL)	71.72	40.20	43.57	26.37	46.77	29.60
Creatinine (mg/dL)	1.37	0.76	1.37	5.33	1.37	5.02
Estimated glomerular filtrate (CKD-EPI 2009) (mL/min/1.73 m^2^)	56.88	23.86	73.79	20.22	71.84	21.33
Sodium (mmol/L)	135.85	4.04	134.91	3.39	135.02	3.48
Potassium (mmol/L)	4.14	0.58	4.20	2.82	4.19	2.66
Chloride (mmol/L)	101.85	3.77	101.13	5.70	101.21	5.52
Total bilirubin (mg/dL)	0.76	0.42	1.64	9.60	1.54	9.05
Aspartate aminotransferase (AST/GOT) (U/L)	72.77	100.68	48.48	36.15	51.20	48.22
Alanine aminotransferase (ALT/GPT) (U/L)	51.83	75.99	42.17	40.45	43.21	45.59
Lactate dehydrogenase (LDH) (U/L)	411.46	201.74	325.42	128.12	335.40	140.97
Albumin (g/dL)	3.53	0.40	3.82	0.38	3.79	0.39
C-reactive protein (mg/dL)	115.47	70.71	78.23	69.85	82.22	70.79
Procalcitonin (ng/mL)	0.81	2.93	0.25	0.92	0.31	1.30
D-dimer (ng/mL)	1500.51	2568.30	1155.00	2318.82	1192.10	2345.08
Fibrinogen (Derived) (mg/dL)	688.53	171.63	667.34	161.70	669.71	162.71
Ratio (TP)	2.03	4.62	1.90	7.06	1.91	6.83
Ratio (TTPA)	29.83	3.87	30.78	5.24	30.67	5.11
pH	7.41	0.07	7.43	0.07	7.42	0.07
pCO_2_ (mmHg)	36.91	9.16	34.75	7.05	35.05	7.39
pO_2_ (mmHg)	64.20	24.83	60.80	23.28	61.27	23.48
Bicarbonate (CO_3_H^−^) (mmol/L)	23.01	4.61	23.07	3.10	23.06	3.34
FIO_2_ (%)	26.86	10.08	24.18	11.32	24.57	11.16
pO_2_/FIO_2_	256.10	86.47	279.83	109.36	276.21	106.32
Arterial/alveolar O_2_ gradient (mmHg)	72.19	55.01	65.13	76.85	66.11	74.11
Lactate (mmol/L)	1.89	1.03	1.72	1.16	1.74	1.15
Days in hospital	20.59	17.44	14.92	18.34	15.54	18.30
Days elapsed between PCR and hospital admission	3.13	5.80	4.00	4.75	3.91	4.87
Number of consolidations	2.57	1.27	2.47	1.55	2.48	1.51
Number of opacities	3.41	1.76	3.18	1.69	3.20	1.69
Curb 65 Scale value	1.63	0.81	0.74	0.81	0.84	0.86
Temperature (°C)	36.87	1.08	36.70	0.94	36.72	0.96
Systolic blood pressure (SBP)	135.41	23.07	131.94	22.62	132.32	22.66
Diastolic blood pressure (DBP)	63.31	16.10	71.92	16.58	70.98	16.72
Heart rate	87.56	19.81	90.90	17.51	90.54	17.78
Respiratory rate	25.32	7.67	23.01	6.95	23.34	7.07
Glasgow Coma Scale value	14.43	1.32	14.86	0.78	14.81	0.87
SOFA scale Value	4.45	1.23	4.07	1.63	4.17	1.54
APACHE-II scale value	12.80	4.69	8.31	3.23	9.53	4.16
Number of COVID-19 vaccine doses	1.00	0.00	1.29	0.46	1.26	0.45

**Table 5:** The final results of accuracy, recall, precision, specificity and kappa are shown in the table.

	Accuracy (%)	Recall	Precision	Specificity	Kappa
SVM	83.32	83.42	82.73	83.22	73.39
BLDA	80.37	80.47	79.78	80.27	71.41
DT	85.52	85.62	84.98	85.42	75.46
GNB	74.82	74.91	74.35	74.73	66.67
KNN	89.24	89.31	89.14	89.18	79.01
RF	95.83	95.92	95.15	95.73	86.32

**Table 6:** The final results of F1 score, MCC, DYI, AUC and AUC (%) are shown in the table.

	F1 Score	MCC	DYI	AUC	AUC (%)
SVM	83.07	73.93	83.32	82	0.82
BLDA	80.12	71.31	80.37	79	0.79
DT	85.29	75.94	85.52	84	0.84
GNB	74.63	65.83	74.82	74	0.74
KNN	89.23	79.48	89.24	89	0.89
RF	95.53	86.83	95.83	95	0.95

## Data Availability

The datasets employed and analyzed in the current study are accessible upon reasonable request from the corresponding author. We do not have the patients’ permission to publish the data collected in this study in open access.

## Abbreviations

The following abbreviations are used in this manuscript:

COVID-19	Coronavirus Disease 19
ICU	Intensive Care Unit
PCR	Polymerase Chain Reaction
AI	Artificial Intelligence
ML	Machine learning
RF	Random Forest
CURB	Confusion, Urea, Respiratory rate, Blood pressure
SOFA	Sequential Organ Failure Assessment
APACHE-II	Acute Physiology and Chronic Health Evaluation
MCV	Mean Corpuscular Volume
PT	Prothrombin time
INR	International normalized Ratio
aPTT	Activated Partial Thromboplastin Time
CKD-EPI	Chronic Kidney Disease Epidemiology Collaboration
ALT/GPT	Alanine Aminotransferase/Glutamate Pyruvate Transaminase
AST/GOT	Aspartate Aminotransferase/Glutamate Oxaloacetate Transaminase
GGT	Gamma-Glutamyl Transferase
LDH	Lactate Dehydrogenase
ROC	Receiver Operating Characteristic
AUC	Area Under the Curve
GNB	Gaussian Naïve Bayes
KNN	K-Nearest Neighbors
BLDA	Bayesian Linear Discriminant Analysis
SMV	Support Vector Machine
DT	Decision Tree
COPD	Chronic Obstructive Pulmonary Disease
IMV	Invasive Mechanical Ventilation
HFNC	High-Flow Nasal Cannula
NIV	Non-Invasive Mechanical Ventilation
ECMO	Extracorporeal Membrane Oxygenation
ARDS	Acute Respiratory Distress Syndrome
SD	Standard Deviation
CRP	C-Reactive Protein
SBP	Systolic Blood Pressure
DBP	Diastolic Blood Pressure
MCC	Mathew’s Correlation Coefficient
DYI	Degenerated Youden’s Index
